# Recombinant PilS: Cloning, Expression and Biochemical Characterization of a Pil-Fimbriae Subunit

**DOI:** 10.3390/microorganisms10061174

**Published:** 2022-06-07

**Authors:** Danielle D. Munhoz, Jessika C. A. Silva, Natalia C. Freitas, Leo K. Iwai, Karina A. Aires, Christiane Y. Ozaki, Cristiane S. Souza, Letícia B. Rocha, Miriam A. Silva, Izabella M. Henrique, Waldir P. Elias, Eneas Carvalho, Ligia Morganti, Rosa M. Chura-Chambi, Roxane M. F. Piazza

**Affiliations:** 1 Laboratório de Bacteriologia, Instituto Butantan, Avenida Vital Brazil, 1500, São Paulo 05503-900, SP, Brazil; dani.dmunhoz@hotmail.com (D.D.M.); jessika.alves@esib.butantan.gov.br (J.C.A.S.); freitas.natalia14@gmail.com (N.C.F.); karaujoaires@gmail.com (K.A.A.); chrisozaki@usp.br (C.Y.O.); crssouza05@gmail.com (C.S.S.); miriam.silva@butantan.gov.br (M.A.S.); izabella.henrique@esib.butantan.gov.br (I.M.H.); waldir.elias@butantan.gov.br (W.P.E.); eneas.carvalho@butantan.gov.br (E.C.); 2 Laboratório de Toxinologia Aplicada, Instituto Butantan, Avenida Vital Brazil, 1500, São Paulo 05503-900, SP, Brazil; leo.iwai@butantan.gov.br; 3 Laboratório Estratégico de Diagnóstico Molecular, Instituto Butantan, Avenida Vital Brazil, 1500, São Paulo 05503-900, SP, Brazil; leticia.rocha@butantan.gov.br; 4 Centro de Biotecnologia, Instituto de Pesquisas Energéticas e Nucleares IPEN-CNEN/SP, São Paulo 05508-000, SP, Brazil; lmorganti@bol.com.br

**Keywords:** adhesin, Pil fimbriae, recombinant protein, cloning, expression, biochemical characterization

## Abstract

Pil-fimbriae is a type IV pili member, which is a remarkably versatile component with a wide variety of functions, including motility, attachment to different surfaces, electrical conductance, DNA acquisition, and secretion of a broad range of structurally distinct protein substrates. Despite the previous functional characterization of Pil, more studies are required to understand the regulation of Pil expression and production, since the exact mechanisms involved in these steps are still unknown. Therefore it is extremely important to have a protein with the correct secondary and tertiary structure that will enable an accurate characterization and a specific antisera generation. For this reason, the aim of this work was to generate potential tools for further investigations to comprehend the mechanisms involved in Pil regulation and its role in pathogenic *E. coli* infections with the obtaining of a precise native-like recombinant PilS and the corresponding antisera. The *pilS* gene was successfully cloned into an expression vector, and recombinant PilS (rPilS) was efficiently solubilized and purified by metal affinity chromatography. Protein characterization analyses indicated that rPilS presented native-like secondary and tertiary structures after the refolding process. The generated anti-rPilS sera efficiently recognized recombinant and native proteins from atypical enteropathogenic *E. coli* strains.

## 1. Introduction

Although bacterial attachment is not enough to establish and maintain an infection, the ability of bacteria to bind to host cell surfaces is an important step in colonization, as this generates a stable platform for multiplication, reducing the host capability of bacterial clearance. Adherence to cell surfaces is not only related to physical bacterial attachment, but also to the activation and delivery of virulence factors, and it may promptly affect host cell signaling that will aid bacterial spreading and survival [1]. Such a virulence factor may allow a more effective bacterial adherence to the intestinal epithelium and/or biofilm formation, which could lead to more serious, chronic and persistent infections [2,3].

Some adhesive organelles known as fimbriae or pili are filamentous, superficial and non-flagellar appendages that may additionally function in phage recognition, DNA transfer, cell aggregation, and cell invasion [4]. The type IV pili are a group of polymeric surface organelles classified as one of the most widespread fimbriae in eubacteria as well as in Archaea [5,6,7,8]. This group of fimbriae is described as long fibrillar structures composed of homopolymers of low molecular weight structural proteins called pilins [9] that are produced from prepilin molecules undergoing cleavage of the leader sequence by a prepilin peptidase [10].

Pil fimbriae is a type IV pili originally described as involved in plasmid conjugation mechanisms in the Salmonella plasmid R64 [11,12]. However, it was further reported as a widespread virulence factor since the genes that comprise the pil operon were also identified in other plasmids in different diarrheagenic *E. coli* pathotypes, as Enteroaggregative *E. coli* (EAEC), Enteropathogenic *E. coli* (EPEC), Enterotoxigenic *E. coli* (ETEC), and Shiga Toxin producing *E. coli* (STEC) [10,13,14,15,16]. In addition to pathotypes, different Pil operon genes, including *pilS* and *pilV* that encode the fimbrial structural protein and the fimbrial adhesin, respectively [17,18,19], were also detected in strains isolated from distinctive geographic regions in different decades, with genes present in more than 20 *E. coli* serotypes [14,16,20]. As described in Salmonella, Pil possibly display a significant function in conjugation mechanisms in *E. coli*, since insertional inactivation of *pilS* reduced conjugal transfer of the carrying plasmid by four orders of magnitude, and its presence was detected in conjugative transfer in the majority of EPEC strains [14,20]. Its role was further associated to biofilm formation on abiotic surfaces and the adherence process, where an *E. coli* K12 derivative carrying a plasmid encoding *pil* gene became an adherent strain, displaying a specific adherence pattern of EAEC pathotype, and inactivation of *pilS* led to almost 75% of adherence reduction to HT29 cells [20]. Despite the indication that the acquisition of a plasmid carrying the *pil* operon could confer to other *E. coli* strains, an enhancement of adherence ability and the influence of the specific adherence pattern on epithelial cells, the precise mechanism involved between Pil and the host target, and whether this structure improves biofilm formation is still unknown.

A complete characterization of the molecular interactions between fimbriae and its receptor is crucial for the understanding of pathogenic mechanisms, as even a small sequence variation or protein conformation alteration may have an effect on fimbriae function. Ideally, fimbriae could be simply purified in their native nature from prototypical strains; however, this scenario may be complicated for several reasons, mainly those related to the availability and fragility regarding fimbrial purification. An efficient and well-applied approach to obtain and study protein function is based on heterologous protein expression, mostly using *E. coli* as host [21]. Since the Pil fimbriae are formed by several subunits, the study of its expression and function is extremely important to obtain a protein with the correct conformational structure. Traditional refolding processes that solubilize proteins in inclusion bodies by denaturation, which are commonly employed protocols, often lead to problems in protein conformation that may impair its characterization [22,23]. For this reason, the aim of this work was to obtain a functional recombinant PilS (rPilS) that is useful for the production of an efficient antibody that will enable further characterization studies of Pil fimbriae in different pathogens.

## 2. Materials and Methods

### 2.1. Strains and Plasmids

Strains and plasmids used in this study are listed in Table 1. Strains BA356, BA558, BA1244 and BA3378 were isolated from the feces of children under five years of age with acute diarrhea in an epidemiological study in Brazil [24]. These strains were characterized as belonging to the atypical EPEC (aEPEC) pathotype due to the presence of the *eae* gene, and the absence of the EAF probe and the virulence genes that characterize the other diarrheagenic *E. coli* pathotypes [25]. BA558, BA1244 and BA3378 harbor the *pilS* gene; while in BA356 this gene is absent [13]. C1096 is an enteroaggregative *E. coli* (EAEC) strain implicated in a neonatal ward outbreak in Serbia [26]. EAEC C1096 encodes Pil fimbriae that contributes to plasmid conjugation, epithelial cell adherence, and adherence to abiotic surfaces [20].

### 2.2. PilS Amplification

Oligonucleotides specific to the *pilS* gene were designed using the Gene Runner^®^ program, based on sequences deposited in the NCBI database (NZ_ABHS01000025.1) from pil operon genes contained in plasmid pSERB1 of atypical EAEC [20] (Table 2). The BamHI and HindIII restriction sites were added to the forward and reverse primers, respectively, to enable further cloning into an expression vector (pET28a). BA558 was first cultivated in lysogenic broth (LB) at 37 °C for 18 h, and later plated in an LB Agar (LBA) medium and incubated at 37 °C for 18 h. Lysates from the bacterial colony were obtained by boiling and ice bath from LBA plates. In Table 2 the primer sequences used on the *pilS* gene amplification are shown.

PCR reactions containing 20 pmol of each of the primers (forward and reverse), 1 U of Taq DNA Polymerase (Invitrogen, Waltham, MA, USA), 1.5 mM magnesium chloride, 200 µM dNTP mix (dATP, dTTP, dCTP and dGTP), 2.5 µL PCR reaction buffer (Invitrogen, Waltham, MA, USA) and sterile ultrapure water for a final volume of 25 µL were subjected to the following amplification cycle: 94 °C/5 min, 30 × 94 °C/1 min, 50 °C/1 min, 72 °C/1 min, and a final extension at 72 °C/7 min. The amplicons were analyzed by 0.8% agarose gel electrophoresis stained with UniSafe Dye (Uniscience, Miami, FL, USA), and photographed with the aid of the AlphaImager™2200 imaging system (Alpha Innotech, San Leandro, CA, USA).

### 2.3. pilS Gene Cloning

In order to obtain recombinant PilS, the corresponding encoding gene was primarily cloned into pGEM-T Easy vector (Promega, Madison, WI, USA), followed by subcloning into the expression vector pET28a (Novagen, Darmstadt, Hesse, Germany).

The *pilS* gene was PCR amplified according to the conditions described above. Cloning into the pGEM-T vector was performed using the pGEM-T Easy Vector Systems kit (Promega, Madison, WI, USA) in a 1:3 ratio (vector: insert) with T4 DNA ligase enzyme (Fermentas, Waltham, MA, USA). The reaction was carried for 1 h at room temperature, followed by incubation at 4 °C for 18 h. The obtained recombinant plasmids were transformed by heat shock into competent *E. coli* JM109 strains.

The pGEM-T cloning confirmation was performed by PCR and restriction enzyme digestion, after plasmid extraction using a PureLink Quick Plasmid Miniprep kit (Invitrogen, Waltham, MA, USA), according to the manufacturer’s recommendations. Restriction analysis using BamHI and HindIII enzymes was performed to confirm the cloning of the insert of interest, as well as to obtain the fragment for further cloning into an expression vector. After digestion and agarose gel electrophoresis, the released fragment was purified from gel using an Illustra GFX PCR DNA and Gel Band Purification kit (GE Healthcare, Chicago, IL, USA), following the manufacturer’s recommendations.

The expression vector pET28a (Novagen, Darmstadt, Hesse, Germany) and the fragment were digested with the restriction enzymes BamHI and HindIII. Cloning was performed using a 1:3 ratio (vector:insert), with T4 DNA ligase enzyme (Fermentas, Waltham, MA, USA). The reaction was incubated at 16 °C for 18 h in a GeneAmp PCR System 9700 thermal cycler (Applied Biosystems, Waltham, MA, USA). Competent *E. coli* JM109 strains were transformed with the obtained construct by heat shock, and recombinant clones were selected after growth in LBA containing 50 μg/mL of kanamycin (Invitrogen, Waltham, MA, USA) at 37 °C for 18 h. The cloning confirmation was performed by PCR and restriction enzyme digestion using BamHI/HindIII enzymes. 

For additional confirmation of in-frame cloning, sequencing of the *pilS* insert cloned into the pET28a vector was carried out at the Human Genome Studies Center of the Biosciences Institute of the University of São Paulo. Each sequencing reaction of plasmid pET28a contained 20 ng of plasmid DNA and 5 pmol/μL of forward and reverse primer from the T7 promoter. Sequencing reactions were performed in an ABI 3730 DNA Analyser (Applied Biosystems, Waltham, MA, USA), according to manufacturer’s protocol, and the obtained results were analyzed based on the *pilS* gene sequence contained in the aEAEC pSERB1 plasmid sequence (NCBI: NZ_ABHS01000025.1), deposited in NCBI databases. The software ClustalW2, Gene Runner^®^, Geneious, and the BLASTn tool from NCBI were applied in the analysis.

### 2.4. Expression of PilS

BL21(DE3) (Novagen, Darmstadt, Hesse, Germany) competent cells transformed with the PilS-pET28a plasmid were inoculated into 500 mL of the rich medium two-fold HKSII [30] containing kanamycin (50 μg/mL) and grown (37 °C, 200 rpm) to an OD_600 nm_ between 2.5 and 3.0., and then isopropyl-b-D-thiogalactoside (IPTG) was added to the concentration of 0.5 mM. The temperature was reduced to 30 °C and the agitation (150 rpm) continued for 16 h. PilS inclusion bodies (IB) isolation was performed as previously described [31]. Aliquots of the PilS-IBs suspension were separated into 1 mL aliquots that were kept in a freezer (−20 °C) until time of use. Analysis of bands in SDS-PAGE for the determination of PilS-IBs purity was performed using the Image J program.

### 2.5. Refolding at High Hydrostatic Pressure (HHP) of PilS-IBs

The PilS-IBs were diluted to a concentration of 1 mg/mL in a refolding buffer (50 mM CAPS pH 11.0, 1 mM EDTA, 0.4 M Arginine and 10 mM DTT). A volume of 1 mL of the suspension of *pilS*-IBs was placed into plastic bags, sealed and introduced into another larger plastic bag and, then vacuum-sealed. The bag was placed inside a pressure vessel (R4-6-40, High Pressure Equipment, Erie, PA, USA) and pressurized at 2.4 kbar for 90 min using oil as a transmission fluid (PS-50, High Pressure Equipment, USA). After decompression, the samples were then centrifuged at 12.000× *g* for 15 min to remove insoluble aggregates and dialyzed against 50 mM Tris–HCl, pH 7.4 buffer. The solution was centrifuged again to remove insoluble aggregates formed during the dialysis process.

### 2.6. Purification of PilS

Dialyzed, refolded PilS was applied to a pre-equilibrated 1 mL HisTrap HP column (GE Healthcare, Chicago, IL, USA) with 50 mM Tris–HCl, pH 7.4 (equilibration buffer), at a flow rate of 1 mL/min. The column was washed with 10 mL of the equilibration buffer and then eluted using an increasing imidazole gradient (0–1 M). Purified *pilS* was dialyzed against 50 mM Tris–HCl, pH 7.4 buffer to remove imidazole.

### 2.7. PilS Biochemical Characterization

#### 2.7.1. In Gel Digestion

The protein band corresponding to PilS at the molecular weight of ~23 kDa was excised from the SDS-polyacrylamide gel and incubated for 3 h in a 50% methanol, 5% acetic acid solution to destain the gel and remove SDS. The supernatant was removed, and the gel was dehydrated with 100% acetonitrile followed by vacuum centrifugation (SpeedVac RVC-2-18/ Alpha 1-2, Christ, Osterode am Harz, Germany). To the dried gel fragment, 10 mM solution of dithiothreitol in 100 mM ammonium bicarbonate was added to reduce disulfide bridges for 30 min at room temperature, followed by incubation in the dark with 50 mM iodoacetamide in 100 mM ammonium bicarbonate for 30 min for alkylation of the SH groups of the cysteine side chains. The gel was washed with 100 mM ammonium bicarbonate and proteins were digested, incubating the gel overnight at 37 °C with 50 μg/mL of trypsin (Sigma-Aldrich, Saint Louis, MO, USA). The digested protein extraction was preceded by incubating the gel in 5% formic acid for 10 min and 5% formic acid in a 50% acetonitrile solution for 10 min. Supernatants were collected in a clean low-binding tube (Axygen, Corning Life Sciences, New York, NY, USA), vacuum dried and resuspended in 0.5% formic acid solution for mass spectrometry analysis. 

#### 2.7.2. Mass Spectrometry and In Silico Analysis

Five microliters of the in gel digested sample were analyzed in an Easy 1000 nano-liquid chromatography system (Thermo Scientific, Bremen, Germany) coupled to an LTQ-Orbitrap Velos mass spectrometer (Thermo Scientific, Bremen, Germany). The peptides were loaded onto an in-house packed C18 (Jupiter 10 μm beads, Phenomenex Inc., Torrance, CA, USA) pre-column (100 μm ID × 360 μm OD) and separated on an in house packed C18 (ACQUA, 3 μm beads, Phenomenex Inc, Torrance, CA, USA) analytical column (75 μm ID × 360 μm OD) on which they were separated over a 35 min gradient using solvent A (0.1% formic acid in water) and solvent B (0.1% formic acid in acetonitrile). The gradient consisted in a constant flow of 200 nL/min with a gradient of 5% to 95% B for 18 min, followed by wash steps with 95% of solvent B for 5 min and 13 min with 5% solvent A. The mass spectrometer was operated in full scan mode, where the top five most intense precursor ions were selected in a data-dependent acquisition mode and fragmentation occurred by collision-induced dissociation (CID) for MS/MS. Data acquisition was performed using Xcalibur 1.4 (Thermo Scientific). Capillary voltage of the nanospray was set to 2.3 kV, the source temperature was set to 250 °C, injection time in ion-trap was set to 100 ms and in the FT-MS it was set to 1000 ms. The MS1 were acquired in FTMS from 300 to 1800 *m*/*z* at a resolution of 30,000, and the spectra of the product ions with the MS2 resolution of 7500. The MS2 was performed in ITMS with CID method at a normalized collision energy of 35.0, isolation width of 2.0 *m*/*z*, default charge state of 2, activation Q of 0.250, and activation time of 10,000, and charge states equal to 1, and unassigned states were rejected. Dynamic exclusion was set to 35 s. Mass spectrometry raw data was converted to mgf using MS Convert software (v.3.0.4445, ProteoWizard, SourceForge) and analyzed in Mascot software (Version 2.6.0, Matrix Science, Boston, MA, USA) with the following settings: cysteine carbamidomethyl as fixed modification, oxidation of methionine as variable modification, trypsin as proteolytic enzyme with 2 allowed miss-cleavages and the tolerance for MS1 and MS2 error was set as 10 ppm and 0.6 Da, respectively. The PilS protein was identified by performing the search using an E. coli database downloaded from Uniprot. The PilS amino acid sequence was investigated in silico employing the software Phyre2 (http://www.sbg.bio.ic.ac.uk/~phyre2/, accessed on 21 March 2022), in which the PDB file was created via prediction, with the exclusion of the non-canonic amino acid U. Then PyMol (PyMOL Molecular Visualization System, DeLano Scientific, San Carlos, CA, USA) and the Jmol program (Jmol: an open-source Java viewer for chemical structures in 3D, http://www.jmol.org/, accessed on 21 March 2022) were employed to predict the structure of recombinant PilS. 

#### 2.7.3. Fluorescence Spectroscopy

A fluorescence analysis was taken with a Cary Eclipse (Varian) Spectrofluorimeter. Data were collected using 1 cm optical path cuvettes, and the measures were performed at a 90° angle relative to the incident light using a 1 s response time and a reading speed of 240 nm/min. The intrinsic fluorescence was collected between 300 and 400 nm, with excitation at 280 nm. For the GdnHCl-induced unfolding procedure, samples of purified PilS were diluted into solutions of guanidine at different concentrations for 2 h, and Trp emission fluorescence measurements were carried out.

#### 2.7.4. Circular Dichroism

The protein’s secondary structure was confirmed after refolding by circular dichroism (CD). The CD spectra were recorded between 183 and 260 nm using a quartz cuvette (0.1 mm path length) in a JASCO J-810 Spectropolarimeter (Jasco Corporation, Tokyo, Japan). After buffer-background subtraction (10 mM sodium phosphate buffer, pH 8.0), the CD data were converted to mean residue ellipticity [θ] units (degree × cm^2^ × dmol^−1^). The results were analyzed on the online server DICHROWEB [32,33,34]. The in silico secondary structure prediction was performed on QiluBio, JPre4 and RaptorX servers. 

### 2.8. Generation of PilS Polyclonal Antibody

Female mice six to eight weeks old were immunized with purified recombinant PilS, and the immunization protocol consisted of one immunization followed by two boosters (30:15:15 days intervals), subcutaneously on the back, in a total volume of 100 µL with purified recombinant protein PilS (10 µg) emulsified in MONTANIDE™ ISA (*v*/*v*) and 0.01 M phosphate buffered saline of pH 7.4 (PBS). Serum was collected before immunization (pre-immune) and 15 days after the administration of the last booster dose and tested by indirect ELISA. The experiments were conducted in accordance with the Ethical Principles in Animal Research, adopted by the Brazilian College of Animal Experimentation, and were approved by the Ethics Committee in Animal Research of the Butantan Institute (CEUA Number 2326140619).

For titration, sera was adsorbed five times against *E. coli* DH5-alpha, and tested from 1:100 to 1:3.276800 in a 96-well MaxiSorp microplate (Nunc, Thermo Fisher Scientific, Waltham, MA, USA) coated with 1 µg/mL of purified recombinant PilS. The reactivity of PilS antisera was also confirmed by immunoblotting analysis. Briefly, 20 µg of recombinant PilS was applied to a 15% SDS-polyacrylamide gel [35,36]. After electrophoresis, the separated proteins were transferred to a nitrocellulose membrane (Nitrocellulose blotting Membrane, Amersham™ Protran™, GE Healthcare Life Sciences, Chicago, IL, USA) at 10 V for 35 min (Trans-blot SD Semi-dry Transfer Cell, Bio-Rad). The membrane was blocked with 1% BSA for 16–18 h and reacted with 1:100 dilution anti-pilS antiserum (1 h). The membrane was then washed and incubated for 1 h with peroxidase goat anti-rabbit IgG (1:5000). After washing, 0.7 mg/mL of diaminobenzidine plus 0.8 µL/mL hydrogen peroxide was added and the reaction was stopped by adding distilled water. Washing steps were done with PBS containing 0.05% Tween-20.

### 2.9. Detection of PilS in Bacterial Cells by Using Polyclonal Antibody

PilS detection was performed as previously described [20]. Briefly, bacterial cells were grown overnight in LB at 37 °C and then incubated in Dulbecco’s Modified Eagle Media (DMEM) containing 2% glucose in a 96-well MaxiSorp microplate (Nunc, Thermo Fisher Scientific, Waltham, MA, USA) in a 1:50 dilution. After 4 h of growth, the medium was removed and non-adherent bacteria were removed by five washes with phosphate-buffered saline 0.01 M pH 7.2 (PBS). Cells were fixed for 15 min with PBS containing 2% glutaraldehyde and 3 mM CaCl_2_ followed by a single washing with PBS. Next procedure is the blocking step with PBS containing 1% bovine serum albumin (BSA) for 16–18 h at 4 °C in a humid chamber. The washing solution used from the blocking step between the subsequent steps was PBS—Tween 0.05% for 3 times.

After the blocking step, the cells were incubated for 1 h at 37 °C with an anti-PilS polyclonal antibody (1:500) followed by goat anti-mouse IgG peroxidase conjugate (1:5000) for 30 min at 37 °C. The reaction was developed using 0.5 mg/mL O-phenylenediamine (OPD; Sigma Aldrich Co., Saint Louis, MO, USA) in addition to 0.5-µL/mL hydrogen peroxide in 0.05 M citrate-phosphate buffer, in the dark at room temperature. The absorbance was measured at wavelength at 492 nm in a Multiskan FC (Thermo Fisher Scientific, Waltham, MA, USA).

## 3. Results

### 3.1. Cloning and Sequencing of PilS

**Figure 1:**
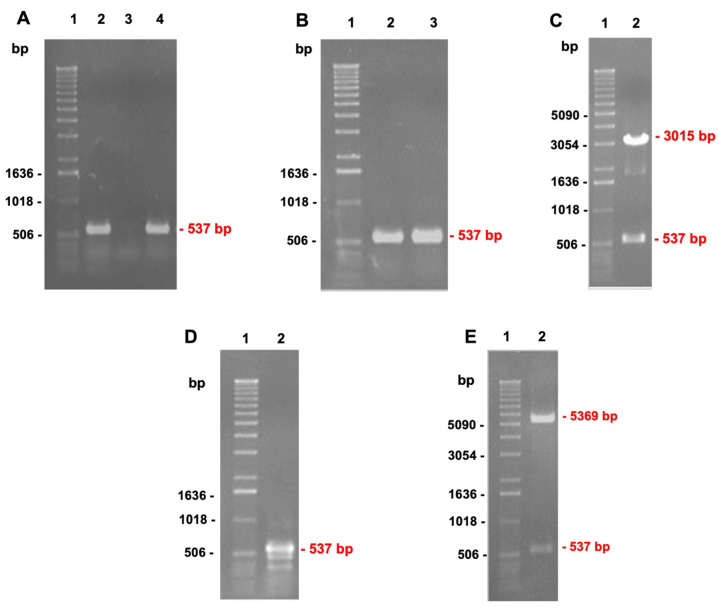
*pilS* cloning steps from aEPEC BA558 strain. Electrophoretic profile in 0.8% agarose gel stained with UniSafe Dye (Uniscience) and visualized on a transilluminator under UV light. (**A**) Amplified fragments after PCR reactions for detection of *pilS* gene in aEPEC BA558 strain: 1. 1 Kb DNA ladder (Invitrogen); 2. EAEC C1096: positive control; 3. *E. coli* C600: negative control; 4. aEPEC BA558 strain; (**B**) Amplified fragments after PCR reactions for *pilS* cloning confirmation in pGEM-T Easy: 1. 1 Kb DNA ladder (Invitrogen); 2. EAEC C1096: positive control; 3. aEPEC BA558 clone (ppilS); (**C**) Amplified fragments from clone digestion with BamHI and HindIII restriction enzymes: 1. 1 Kb DNA ladder (Invitrogen); 2. ppilS clone; (**D**) Amplified fragments in PCR reactions for *pilS* cloning confirmation into pET28a: 1. 1 Kb DNA Ladder (Invitrogen); 2. ppilS clone; (**E**) Amplified fragments from clone digestion with BamHI and HindIII restriction enzymes after pET28a cloning: 1. 1 Kb DNA ladder (Invitrogen); 2. ppilS clone.

**Figure 2:**
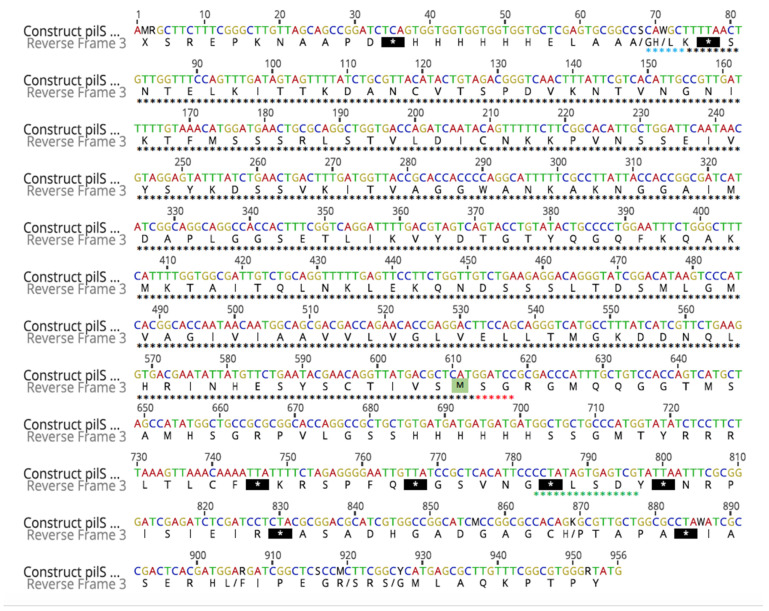
Sequencing of *pilS* constructs in expression vector pET28a. Geneious plus software was applied to analyze and identify the sequences in the construct. The *pilS* sequence is marked with a black asterisk; BamHI and HindIII enzymes sequence are highlighted with red and blue asterisks, respectively; T7 promoter is indicated with green asterisks. In the *pilS* sequence the start codon is highlighted with a green square and the stop codon with a black square.

The presence of *pilS* in an EPEC strain BA558 was confirmed by PCR (Figure 1A). This amplicon was further cloned in a pGEM-T vector and transformed into *E. coli* JM109. The correct cloning was confirmed by PCR with specific *pilS* primers and restriction enzyme analysis (Figure 1B,C). The next step was the cloning of that insert into the pET28a expression vector. This second cloning was also confirmed by PCR with specific *pilS* primers and restriction enzyme analysis (Figure 1D,E). To ensure that the fragment was inserted into the correct frame, the construct was fully sequenced. After an identity analysis with BLASTn tool from NCBI, 100% identity was determined with *pilS* of pSERB1 sequence, and a correct frame with Geneious software (Figure 2).

### 3.2. Solubilization and Refolding at HHP of PilS

**Figure 3:**
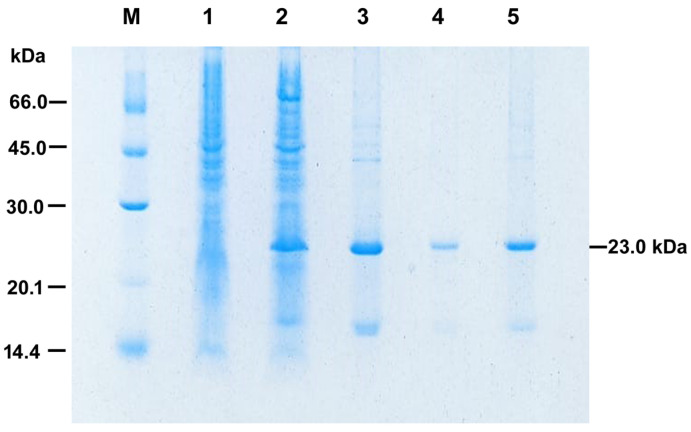
Expression, refolding at high pressure (HHP) and purification of rPilS analyzed by SDS-PAGE (15%). M: molecular mass standards; Lane 1: crude extract of *E. coli* cells before the addition of IPTG; Lane 2: crude extract of cells induced with 0.5 mM IPTG for 16 h at 30 °C; Lane 3: purified rPilS-IBs; Lane 4: soluble rPilS after refolding at high pressure (HHP) and Lane 5: rPilS purified by immobilized metal affinity chromatography.

In order to obtain the production of high levels of recombinant PilS (rPilS), we used the rich medium two-fold HKSII [30]. Bacterial lysis was followed by washing the insoluble IBs with sodium deoxycholate detergent in order to reduce bacterial contaminants. We obtained PilS-IBs with purity greater than 80% as determined by analysis of the rPilS-IBs band on SDS-PAGE using the Image J program (Figure 3).

Combinations of HHP, alkaline pH (11.0), and the addition of additives (0.4 M arginine and 10 mM DTT) were used in order to solubilize IBs from rPilS expressed in *E. coli* (*pilS*-IBs). HHP is an efficient technique for the solubilization and renaturation of proteins produced as IBs [31]. rPilS was efficiently solubilized at high pressure and alkaline pH, and did not suffer reaggregation by decompression and pH reduction to 7.4 by dialysis. We also established a purification process by immobilized metal affinity chromatography using the refolded rPilS at HHP. The protein eluted at a 50% imidazole concentration (0.5 M) was dialyzed against a 50 mM Tris–HCl, pH 7.4 buffer and analyzed by SDS-PAGE. A component of relative electrophoretic mobility of 23.0 kDa corresponds to purified recombinant rPilS (Figure 3).

### 3.3. rPilS Identification

**Figure 4:**
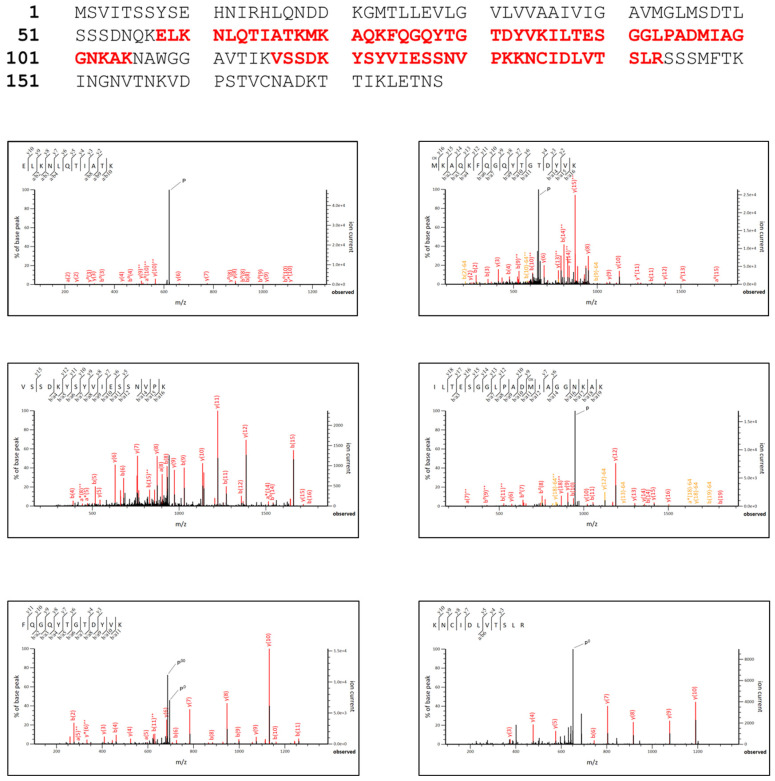
Mass spectrometry identification by shotgun proteomics analysis. Identified peptides in rPilS are shown in the top panel and each corresponding peptide spectra is shown below. Fragment -b and -y ions of each peptide identified in MS/MS are shown in the top left corner of the spectra. P = parent ion. P^0^ = parent ion-H_2_O, P^00^ = parent ion-2H_2_O, * = -NH_3_, ^0^ = -H_2_O, ^++^ = +2 charge, OX = oxidation (of Methionine).

To confirm the production of recombinant rPilS and its correct translation, a SDS/PAGE band was excised after purification and submitted to mass spectrometry analysis. We identified by trypsin digestion peptide (ELKNLQTIATKMKAQKFQGQUTGTDYVKILTESGGLPADMIAGGNKAKVSSDKYSYVIESSNVPKKNCIDLVTSLR) (Figure 4) with a score of 494, coverage of rPilS protein sequence of about 42% that corresponded to *E. coli* PilS, confirming its correct expression and purification.

**Figure 5:**
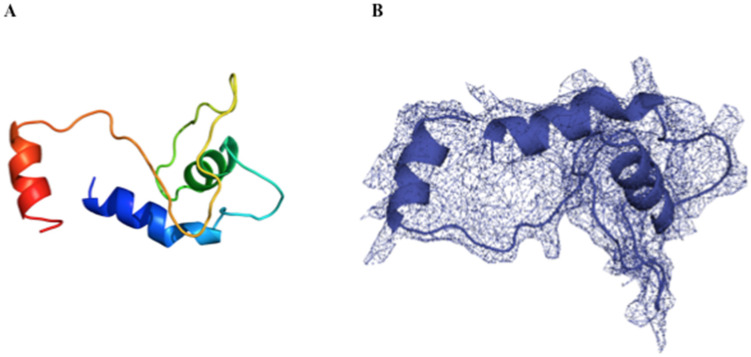
(**A**) Three-dimensional representation in sticks of recombinant PilS fimbriae using Jmol, a spectrum tool in the mode rainbow with the blue representing the N-terminus ascending until reaching a red color at the C-terminus; (**B**) This mesh is the prediction of the electron density map, where the protein is embedded in the mesh.

The tridimensional structure was built using sets of modeling mode in intense (Figure 5A,B). This analysis resulted in high sequence identity (99.2%) with the coverage of 100% employing the information as a template from the pili subunit of the pilin family, therefore confirming the accuracy of this prediction.

### 3.4. rPilS Structure

**Figure 6:**
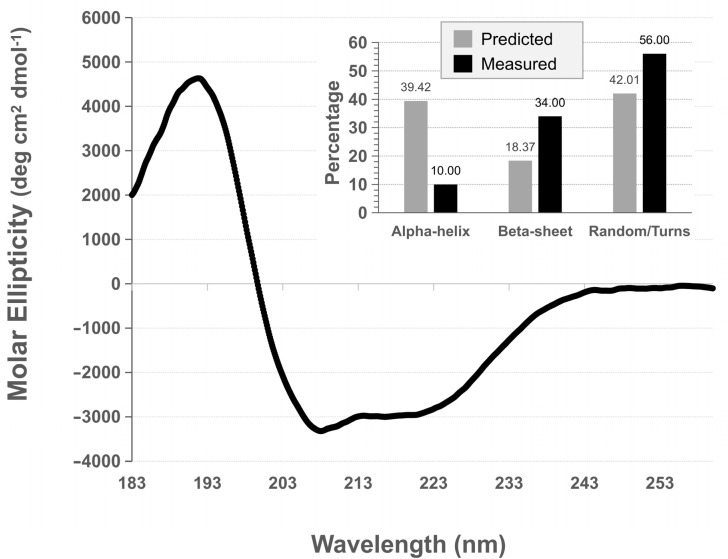
Analysis of secondary structure using circular dichroism. Circular dichroism spectrum and percentages of each class of secondary structure, according to in silico prediction and as measured by circular dichroism after deconvolution.

Circular dichroism analysis showed that PilS has regular secondary structures (Figure 6). Using three independently obtained rPilS samples and three algorithms to infer the secondary structures percentages-based on circular dichroism spectrum (Figure 6) deconvolution, we obtained a mean of 9.30% (±3.08) alpha helix, 32.87% (±6.31) beta-sheet and 56.83 (±7.28) of random/turns structures. On the other hand, the mean percentage of secondary structure, according to three in silico predictions, was 39.42% (±2.21) alpha helix, 18.37% (±2.12) beta-sheet and 42.01 (±3.70) of random/turn structures (Figure 6). Thus, some difference was detected between the secondary structure percentages measured by circular dichroism than those predicted by in-silico algorithms, especially a lower percentage of alpha-helix and a higher percentage of beta-sheets on deconvoluted data when compared to in silico predictions.

**Figure 7:**
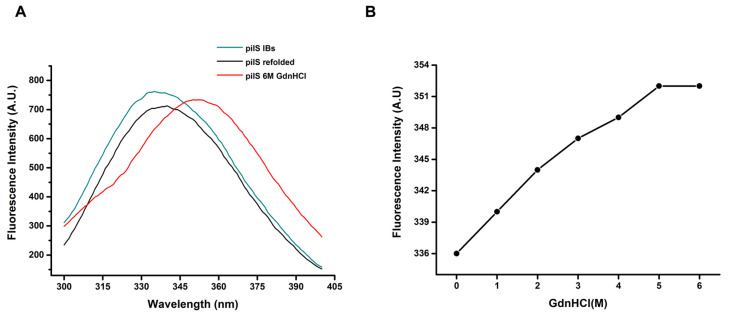
Fluorescence analyses of recombinant rPilS. (**A**) Tryptophan fluorescence emission spectra of rPilS; IBs, rPilS refolded and *pilS* denaturated with 6 M GdnHCl. (**B**) rPilS λ maximum shift induced by rising GdnHCl concentrations.

rPilS contains one tryptophan residue within its structure, which makes the intrinsic tryptophan fluorescence a potential marker of alterations in the tertiary structure. The tryptophan fluorescence emission spectrum of refolded rPilS exhibits a maximal emission wavelength at 337 nm (λ maximum) when excited at 280 nm, which indicates that tryptophan residue is at least partially retained in the hydrophobic core. An interesting finding is the similarity of the rPilS-IBs and refolded rPilS (λ maximum values. (Figure 7A). This result suggests that both proteins present a native-like tertiary structure. The observations that IBs present a native-like tertiary structure have also been described for other proteins [37,38].

In order to assess the maintenance of PilS tertiary structures, we monitored the structural alterations of this protein submitted to unfolding treatments. Shifts to red in the spectrum and increases in intensity of fluorescence are observed by the increase of concentrations of the GdnHCl denaturing agent. rPilS starts to unfold at 1 M GdnHCl (λ maximum 340 nm), continues to unfold at 3M GdnHCl (λ maximum 347 nm) and reaches the highest unfolded state at about 6 M GdnHCl, leading to a maximum emission at 352, which is indicative of protein unfolding by Trp exposure to a hydrophilic environment (Figure 7B). These results suggest that rPilS refolded at HHP has a tertiary structure and its structure is lost when subjected to denaturation by chaotropic agents.

### 3.5. rPilS Recovered by HHP Generates a Polyclonal Antibody Able to Recognize Strains Harboring PilS

**Figure 8:**
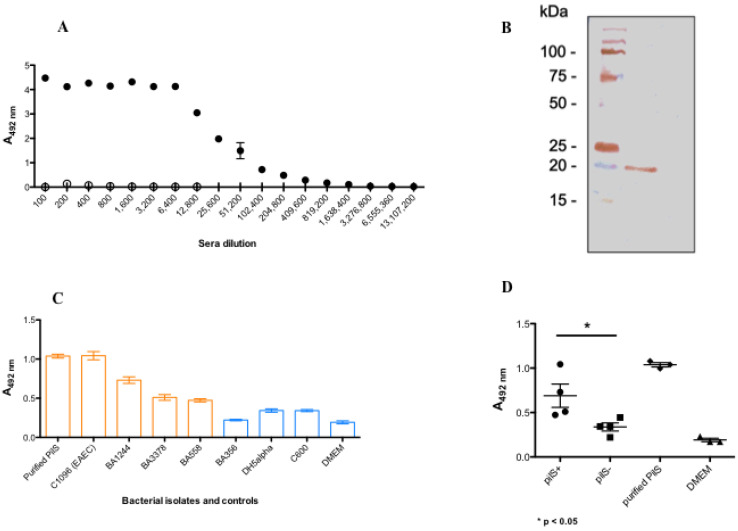
ELISA and immunoblotting of mice anti-rPilS serum for detection of PilS. (**A**) Mice pre immune (empty dot) and anti-rPilS (black dot) sera; (**B**) Nitrocellulose membrane containing protein fractions of rPilS were incubated with 1:100 dilution of PilS antiserum followed by peroxidase goat anti-rabbit IgG (1:5000); the reaction was revealed with DAB + H_2_O_2_. Precision Plus Protein Standards (Kaleidoscope # 1610375, Bio-Rad) (**C**) ELISA for PilS detection in bacteria cells: aEPEC BA1244, BA3378, BA558 and aEAEC (C1096) strains (orange bars). BA356, *E. coli* DH5α and *E. coli* C600 were employed as *pilS*- strains (blue bars). Purified rPilS protein (orange bar) and DMEM (blue bar) were employed as positive and negative controls, respectively. The reaction was carried out with a rPilS polyclonal antibody (1:500) and goat anti-mouse IgG peroxidase conjugate (1:5000); (**D**) comparative analyses of the absorbance values of means were analyzed by One-Way Analysis of Variance comparing *pilS*+, *pilS*-, positive and negative controls (*p* = 0.0003), and between *pilS*+ and *pilS*- groups the means were significantly different (*p* = 0.044). These data are triplicates of two independent experiments.

High titers of antibodies were observed after mice immunization with rPilS, using it as a coating antigen. Considering one optical density (1 OD) the antibody titer was 1:51,200, and applying the reaction cut off of 0.124 the titer was 1:204,800 (Figure 8A). The sera also recognized the recombinant purified PilS by immunoblotting, a protein presenting an electrophoretic mobility of 20 kDa (Figure 8B). Therefore, the generated antibody was employed in an ELISA assay using a microplate coated with the bacterial cells grown for 4 h, the absorbed sera was able to recognize aEPEC strains harboring *pilS* (*pilS*+) as well the Pil prototype EAEC strain (C1096), and the purified rPilS protein. The other *E. coli* strains devoid of *pilS* (*pilS*-), employed as negative control of the assay (C600, DH5α, and BA356), showed results similar to DMEM (Figure 8C). The recognition of this serum was specific to *pilS*+ strains and the differences between the two groups (*pilS*+, *pilS*- and non-pathogenic strains) were statistically significant (*p* = 0.044) (Figure 8D).

## 4. Discussion

In pathogenic *E. coli*, Pil was first described in an EAEC strain denominated C1096 that caused a neonatal diarrhea outbreak in Serbia [26]. In this strain, Pil was necessary for optimal conjugation of a high-molecular weight plasmid (pSERB1), adherence and biofilm formation. Of note, the genes encoding Pil fimbriae were found in approximately 10% of EAEC strains [20]. In a previous study from our group, the *pilS* gene was detected in 8.3% of atypical EPEC strains, a subgroup of EPEC lacking the EAF plasmid [13].

Furthermore, the presence of the *pilS* was searched in a large collection of pathogenic groups of *E. coli* [14], detecting the presence of *pil* genes in all DEC pathovars and uropathogenic *E. coli* (UPEC) strains. However, whether these positive strains carry a complete *pil* operon and whether Pil has a role as a possible virulence factor in these strains is yet to be determined [14].

The development of tools that enable PilS production for in vitro studies are critical for the determination of its biological activity, expression kinetic, and the singular properties of this molecule. Therefore, the aim of this work was to obtain a recombinant PilS protein that could be used for protein characterization, as well as to be used for generation of an anti-PilS serum with high specificity and sensitivity.

In the present study we sequenced and cloned in an expression vector the *pilS* gene present in the aEPEC strain BA558. Traditional approaches for protein refolding from aggregates typically involve solubilizing IBs in concentrated solutions of chaotropes such as GdnHCl or urea, which also results in essentially complete protein unfolding and exposure of hydrophobic residues. There is often significant amount of protein reaggregation when the denaturing reagent is removed, which results in a low recovery yield [39]. HHP is a mild method that efficiently solubilizes protein aggregates which prevents complete denaturation and exposure of hydrophobic patches [40,41]. Alkaline pH breaks intermolecular bonds by electrostatic repulsion [42]. The association of both processes can solubilize protein aggregates without denaturing the proteins present in these structures, maintaining secondary and tertiary structures similar to the one present in native proteins, which are usually present in IBs. Recent studies developed by Morganti and Chura-Chambi demonstrated the efficiency of HHP associated with alkaline pH to solubilize dengue proteins and the non-structural proteins Zika (NS1) expressed as IBs [38,43]. In these studies, we demonstrate that the association of HHP (2.4 kbar) with alkaline conditions (pH 11) allowed the dissociation of aggregates into rPilS that does not reaggregate by decompression and the decreasing of the pH. Furthermore, the presence of arginine (0.4 M), which was shown to have effects during the refolding process, such as improving the solubility and inhibiting protein aggregation [44], was essential to increase the solubilization of rPilS-IBs aggregates. The results obtained indicate that the association of HHP with pH alkaline pH represents a milder alternative for solubilization of rPilS-IBs to maintain the native-like structure and minimize reaggregation. In this work we successfully employed HHP to obtain the major structural subunit of Pil fimbriae, PilS, and to allow its characterization. This protein was confirmed by mass spectrometry with a score of 494, and coverage of the rPilS protein sequence of about 42% that corresponded to PilS, confirming its correct expression and purification. The recombinant protein presented secondary structures, although the measured percentage of structures was not similar to the predicted one. We were unable to affirm if these differences are caused by the fact that the recombinant protein is only partially structured or if the actual structure of the protein is divergent from that predicted by in silico procedures. Some level of tertiary structure was also detected. A tridimensional structure analysis resulted in high sequence identity and coverage of the pili subunit of the pilin family, confirming the accuracy of this prediction. We highlight that antibodies produced against rPils recognized the native protein, suggesting that the structure obtained by refolding resembles the native one, as well as that the protein is a recombinant construction that has a 6xHis-tag, which could give it some non-usual structure, which may deviate from the predicted one. According to the obtained results the recombinant protein possesses a native-like sequence, structure, and conformation, which lead us to conclude that it possesses a native-like function as well. It is important to mention that PilS is the major structural subunit of Pil fimbriae [19]; therefore, this is the main external subunit that connects bacterial membrane to the adhesin subunit (PilV), which actually possesses an adhesive domain to bind to unknown host receptors. The expression of functional pili requires the presence of several accessory genes involved in processing the precursor (prepilin) subunit, secretion across the cell envelope, anchorage, and polymerization of subunits into the pilus organelle [9,45].

Many studies on bacterial pathogenesis are based on the search for virulence determinants using only the molecular techniques to detect the presence of the corresponding encoding genes. However, the phenotypic characterization of these strains is very important, since the expression of genes related to virulence is what determines the pathogenic potential of these isolates [46,47,48,49]. Consequently, a specific and sensitive serum to detect Pil is an important tool for studies about the role of these fimbriae in pathogenesis. An anti-rPilS serum was obtained in this study using the rPilS produced. To avoid cross-reactivity with other protein components besides rPilS, the *E. coli* DH5-α strain was used in immune serum adsorption. By adsorbing the obtained serum, we expected to select only antibodies specific against the protein of interest, thus minimizing possible nonspecific recognitions. As observed in our enzyme immunoassays, the produced anti-rPilS serum was capable of recognizing both the purified protein and distinguishing between *pilS*+ and *pilS*- strains, thus becoming a powerful tool for Pil characterization.

The presence of Pil fimbriae genes in a wide range of pathogenic bacteria indicates that more studies are required to better characterize the processes involved in Pil expression, regulation, and its biological role in host-pathogen interaction. Therefore, the herein obtained recombinant PilS, with correct sequence and conformation, allowed the production of anti-PilS sera capable of detecting the fimbriae produced by pathogenic *E. coli* strains. This will enable further studies regarding Pil as a virulence factor, and may also provide attractive targets for new therapeutic and diagnostic research for pathogenic *E. coli*.

## Figures and Tables

**Table 1:** Strains and plasmid used in the present study.

**Strains**	**Characteristic**	**Reference**
BA558	aEPEC *pilS*+	[13]
BA1244	aEPEC *pilS*+	[13]
BA3378	aEPEC *pilS*+	[13]
BA356	aEPEC *pilS*-	[13]
C600	Non-pathogenic *E. coli*–Negative control	[27]
DH5alpha	Non-pathogenic *E. coli*–Negative control	[27]
C1096	aEAEC	[20,26]
JM109	*E. coli* strain used for cloning and plasmid maintenance	[28]
BL21 (DE3)	*E. coli* strain used as expression host	[29]
**Plasmids**	**Characteristic**	**Reference**
pGEM-T	Replication vector	Promega, Madison, WI, USA
pET28a	Expression vector	Novagen, Darmstadt, Hesse, Germany

**Table 2:** *pilS* primers employed in this study, corresponding amplicon and annealing temperature.

Primer	Annealing Temperature	Fragment
(F) GGATCCATGAGCGTCATAACCTGTTC	50 °C	537 pb
(R) AAGCTTTTAACTGTTGGTTTCCAGTTT		

The underlined sequences correspond to the BamHI and HindIII restriction sites in forward (F) and reverse (R) primers, respectively.

## Data Availability

Some results of the generated datasets for this study can be found in the https://teses.usp.br/teses/disponiveis/42/42135/tde-20092012-095345/pt-br.php. doi:10.11606/D.42.2012.tde-20092012-095345 (accessed on 16 April 2022). Other results are available on request from the corresponding authors.

## References

[1] Kline K.A., Fälker S., Dahlberg S., Normark S., Henriques-Normark B. (2009). Bacterial adhesins in host-microbe interactions. Cell Host Microbe.

[2] DuPont H.L. (2016). Persistent Diarrhea: A Clinical Review. JAMA.

[3] Costerton J.W., Stewart P.S., Greenberg E.P. (1999). Bacterial biofilms: A common cause of persistent infections. Science.

[4] Proft T., Baker E.N. (2009). Pili in Gram-negative and Gram-positive bacteria—Structure, assembly and their role in disease. Cell. Mol. Life Sci..

[5] Giltner C.L., Nguyen Y., Burrows L.L. (2012). Type IV pilin proteins: Versatile molecular modules. Microbiol. Mol. Biol. Rev..

[6] Albers S.V., Pohlschröder M. (2009). Diversity of archaeal type IV pilin-like structures. Extremophiles.

[7] Alphonse S., Durand E., Douzi B., Waegele B., Darbon H., Filloux A., Voulhoux R., Bernard C. (2010). Structure of the *Pseudomonas aeruginosa* XcpT pseudopilin, a major component of the type II secretion system. J. Struct..

[8] Aroeti B., Friedman G., Zlotkin-Rivkin E., Donnenberg M.S. (2012). Retraction of enteropathogenic *E. coli* type IV pili promotes efficient host cell colonization, effector translocation and tight junction disruption. Gut Microbes.

[9] Strom M.S., Lory S. (1993). Structure-function and biogenesis of the type IV pili. Annu. Rev Microbiol..

[10] Collyn F., Léty M.A., Nair S., Escuyer V., Ben Younes A., Simonet M., Marceau M. (2002). *Yersinia pseudotuberculosis* harbors a type IV pilus gene cluster that contributes to pathogenicity. Infect. Immun..

[11] Kim S.R., Komano T. (1997). The plasmid R64 thin pilus identified as a type IV pilus. J. Bacteriol..

[12] Ishiwa A., Komano T. (2004). PilV adhesins of plasmid R64 thin pili specifically bind to the lipopolysaccharides of recipient cells. J. Mol. Biol..

[13] Munhoz D.D., Nara J.M., Freitas N.C., Moraes C.T.P., Nunes K.O., Yamamoto B.B., Vasconcellos F.M., Martínez-Laguna Y., Girón J.A., Martins F.H. (2018). Distribution of Major Pilin Subunit Genes Among Atypical Enteropathogenic *Escherichia coli* and Influence of Growth Media on Expression of the *ecp* Operon. Front. Microbiol..

[14] Garcia B.G., Castro F.S., Vieira M.A.M., Girão D.M., Uenishi L.T., Cergole-Novella M.C., Dos Santos L.F., Piazza R.M.F., Hernandes R.T., Gomes T.A.T. (2019). Distribution of the pilS gene in *Escherichia coli* pathovars, its transfer ability and influence in the typical enteropathogenic *E. coli* adherence phenotype. Int. J. Med. Microbiol..

[15] Zhang X.L., Tsui I.S., Yip C.M., Fung A.W., Wong D.K., Dai X., Yang Y., Hackett J., Morris C. (2000). *Salmonella enterica* serovar typhi uses type IVB pili to enter human intestinal epithelial cells. Infect. Immun..

[16] Srimanote P., Paton A.W., Paton J.C. (2002). Characterization of a novel type IV pilus locus encoded on the large plasmid of locus of enterocyte effacement-negative Shiga-toxigenic Escherichia coli strains that are virulent for humans. Infect. Immun..

[17] Komano T. (1999). Shufflons: Multiple inversion systems and integrons. Annu. Rev. Genet..

[18] Sakai D., Komano T. (2002). Genes required for plasmid R64 thin-pilus biogenesis: Identification and localization of products of the pilK, pilM, pilO, pilP, pilR, and pilT genes. J. Bacteriol..

[19] Yoshida T., Furuya N., Ishikura M., Isobe T., Haino-Fukushima K., Ogawa T., Komano T. (1998). Purification and characterization of thin pili of IncI1 plasmids ColIb-P9 and R64: Formation of PilV-specific cell aggregates by type IV pili. J. Bacteriol..

[20] Dudley E.G., Abe C., Ghigo J.M., Latour-Lambert P., Hormazabal J.C., Nataro J.P. (2006). An IncI1 plasmid contributes to the adherence of the atypical enteroaggregative Escherichia coli strain C1096 to cultured cells and abiotic surfaces. Infect. Immun..

[21] Rosano G.L., Ceccarelli E.A. (2014). Recombinant protein expression in *Escherichia coli*: Advances and challenges. Front. Microbiol..

[22] Rathore A.S., Bade P., Joshi V., Pathak M., Pattanayek S.K. (2013). Refolding of biotech therapeutic proteins expressed in bacteria: Review. J. Chem. Technol. Biotechnol..

[23] Baeshen M.N., Al-Hejin A.M., Bora R.S., Ahmed M.M.M., Ramadan H.A.I., Saini K.S., Baeshen N.A., Redwan E.M. (2015). Production of Biopharmaceuticals in E. coli: Current Scenario and Future Perspectives. J. Microbiol. Biotechnol..

[24] Bueris V., Sircili M.P., Taddei C.R., dos Santos M.F., Franzolin M.R., Martinez M.B., Ferrer S.R., Barreto M.L., Trabulsi L.R. (2007). Detection of diarrheagenic *Escherichia coli* from children with and without diarrhea in Salvador, Bahia, Brazil. Mem. Inst. Oswaldo Cruz.

[25] Abe C.M., Trabulsi L.R., Blanco J., Blanco M., Dahbi G., Blanco J.E., Mora A., Franzolin M.R., Taddei C.R., Martinez M.B. (2009). Virulence features of atypical enteropathogenic *Escherichia coli* identified by the eae(+) EAF-negative stx(-) genetic profile. Diagn. Microbiol. Infect. Dis..

[26] Cobeljić M., Miljković-Selimović B., Paunović-Todosijević D., Velicković Z., Lepsanović Z., Zec N., Savić D., Ilić R., Konstantinović S., Jovanović B. (1996). Enteroaggregative *Escherichia coli* associated with an outbreak of diarrhoea in a neonatal nursery ward. Epidemiol. Infect..

[27] Sambrook J., Fritsch E.R., Maniatis T. (1989). Molecular Cloning: A Laboratory Manual.

[28] Hanahan D., Glover D.M. (1985). Techniques for transformation of *E. coli*. DNA Cloning: A Practical Approach.

[29] Studier F.W., Moffatt B.A. (1986). Use of bacteriophage T7 RNA polymerase to direct selective high-level expression of cloned genes. J. Mol. Biol..

[30] Jensen E.B., Carlsen S. (1990). Production of recombinant human growth hormone in *Escherichia coli*: Expression of different precursors and physiological effects of glucose, acetate, and salts. Biotechnol. Bioeng..

[31] Chura-Chambi R.M., Cordeiro Y., Malavasi N.V., Lemke L.S., Rodrigues D., Morganti L. (2013). An analysis of the factors that affect the dissociation of inclusion bodies and the refolding of endostatin under high pressure. Process. Biochem..

[32] Whitmore L., Wallace B.A. (2008). Protein secondary structure analyses from circular dichroism spectroscopy: Methods and reference databases. Biopolymers.

[33] Whitmore L., Wallace B.A. (2004). Dichroweb: An online server for protein secondary structure analyses from circular dichroism spectroscopic data. Nucleic Acids Res..

[34] Lobley A., Whitmore L., Wallace B.A. (2002). DICHROWEB: An interactive website for the analysis of protein secondary structure from circular dichroism spectra. Bioinformatics.

[35] Laemmli U.K. (1970). Cleavage of structural proteins during the assembly of the head of bacteriophage T4. Nature.

[36] Studier F.W. (1973). Analysis of bacteriophage T7 early RNAs and proteins on slab gels. J. Mol. Biol..

[37] Chura-Chambi R.M., Prieto-da-Silva A.R.B., Di Lela M.M., Oliveira J.E., Abreu P.E.A., Meireles L.R., de Andrade H.F., Morganti L. (2022). High level SARS-CoV-2 nucleocapsid refolding using mild condition for inclusion bodies solubilization: Application of high pressure at pH 9.0. PLoS ONE.

[38] Chura-Chambi R.M., da Silva C.M.R., Pereira L.R., Bartolini P., Ferreira L.C.S., Morganti L. (2019). Protein refolding based on high hydrostatic pressure and alkaline pH: Application on a recombinant dengue virus NS1 protein. PLoS ONE.

[39] Singh S.M., Sharma A., Upadhyay A.K., Singh A., Garg L.C., Panda A.K. (2012). Solubilization of inclusion body proteins using n-propanol and its refolding into bioactive form. Protein Expr. Purif..

[40] Crisman R.L., Randolph T.W. (2009). Refolding of proteins from inclusion bodies is favored by a diminished hydrophobic effect at elevated pressures. Biotechnol. Bioeng..

[41] Silva J.L., Oliveira A.C., Vieira T.C., de Oliveira G.A., Suarez M.C., Foguel D. (2014). High-pressure chemical biology and biotechnology. Chem. Rev..

[42] Yongsawatdigul J., Park J.W. (2004). Effects of alkali and acid solubilization on gelation characteristics of rockfish muscle proteins. J. Food Sci..

[43] da Silva C.M.R., Chura-Chambi R.M., Ramos Pereira L., Cordeiro Y., de Souza Ferreira L.C., Morganti L. (2018). Association of high pressure and alkaline condition for solubilization of inclusion bodies and refolding of the NS1 protein from zika virus. BMC Biotechnol..

[44] Arakawa T., Ejima D., Tsumoto K., Obeyama N., Tanaka Y., Kita Y., Timasheff S.N. (2007). Suppression of protein interactions by arginine: A proposed mechanism of the arginine effects. Biophys. Chem..

[45] Hobbs M., Mattick J.S. (1993). Common components in the assembly of type 4 fimbriae, DNA transfer systems, filamentous phage and protein-secretion apparatus: A general system for the formation of surface-associated protein complexes. Mol. Microbiol..

[46] Wu H.-J., Wang A.H.-J., Jennings M.P. (2008). Discovery of virulence factors of pathogenic bacteria. Curr. Opin. Chem. Biol..

[47] Hernandes R.T., Velsko I., Sampaio S.C., Elias W.P., Robins-Browne R.M., Gomes T.A., Girón J.A. (2011). Fimbrial adhesins produced by atypical enteropathogenic *Escherichia coli* strains. Appl. Environ. Microbiol..

[48] Thomas M.S., Wigneshweraraj S. (2014). Regulation of virulence gene expression. Virulence.

[49] Bakour S., Sankar S.A., Rathored J., Biagini P., Raoult D., Fournier P.E. (2016). Identification of virulence factors and antibiotic resistance markers using bacterial genomics. Future Microbiol..

